# Bile microbiota in primary sclerosing cholangitis: Impact on disease progression and development of biliary dysplasia

**DOI:** 10.1371/journal.pone.0182924

**Published:** 2017-08-10

**Authors:** Pedro Pereira, Velma Aho, Johanna Arola, Sonja Boyd, Kalle Jokelainen, Lars Paulin, Petri Auvinen, Martti Färkkilä

**Affiliations:** 1 Institute of Biotechnology, University of Helsinki, Helsinki, Finland; 2 University of Helsinki, Department of Pathology, Helsinki University Hospital, Helsinki, Finland; 3 University of Helsinki and Clinic of Gastroenterology, Helsinki University Hospital, Helsinki, Finland; Texas A&M University, UNITED STATES

## Abstract

**Objective:**

The etiopathogenesis and risk for development of biliary neoplasia in primary sclerosing cholangitis (PSC) are largely unknown. Microbes or their metabolites have been suggested to play a role. To explore this potential microbial involvement, we evaluated the differences in biliary microbiota in PSC patients at an early disease stage without previous endoscopic retrograde cholangiography (ERC) examinations, advanced disease stage, and with biliary dysplasia or cholangiocarcinoma.

**Design:**

Bile samples from the common bile duct were collected from 46 controls and 80 patients with PSC during ERC (37 with early disease, 32 with advanced disease, and 11 with biliary dysplasia). DNA isolation, amplification, and Illumina MiSeq sequencing were performed for the V1-V3 regions of the bacterial 16S rRNA gene.

**Results:**

The most common phyla found were Bacteroidetes, Firmicutes, Proteobacteria, Fusobacteria, and Actinobacteria. The most common families were Prevotellaceae, Streptococcaceae, Veillonellaceae, Fusobacteriaceae, and Pasteurellaceae, and the most common genera were *Prevotella*, *Streptococcus*, *Veillonella*, *Fusobacterium*, and *Haemophilus*. The bacterial communities of non-PSC subjects and early stage PSC patients were similar. Alpha diversity was lower in patients with biliary dysplasia/cholangiocarcinoma than in other groups. An increase in *Streptococcus* abundance was positively correlated with the number of ERC examinations. *Streptococcus* abundance was also positively correlated with an increase in disease severity, even after controlling for the number of ERC examinations.

**Conclusions:**

Our findings suggest that the aetiology of PSC is not associated with changes in bile microbial communities, but the genus *Streptococcus* may play a pathogenic role in the progression of the disease.

## Introduction

Primary sclerosing cholangitis (PSC) is a chronic inflammatory liver disease leading to strictures of the intra- and extrahepatic bile ducts and finally to cholestasis and secondary biliary cirrhosis [1]. The inflammation is associated with increased proliferation of biliary epithelial cells and a markedly increased risk of development of biliary dysplasia and cholangiocarcinoma [2]. The etiopathogenesis of PSC is unknown, but the disease is considered to be a heterogeneous disorder influenced by genetic, immunologic and environmental factors [3]. PSC is frequently associated with inflammatory bowel disease (IBD) [3], and it has been suggested that microbiota and microbial metabolites or derivatives, for example pathogen-associated molecular patterns (PAMPs) such as lipopolysaccharide, lipoteichoic acid, and peptidoglycan, could play a role in the etiopathogenesis of the disease [4–7]. The association between PSC and IBD could be due to increased enterohepatic circulation of PAMPs (“leaky gut”), or abnormal PAMPs that result from changes in the enteric bacterial community, which has been described in IBD [8–10], or an inappropriate innate immune response to normal levels of enterohepatically circulated PAMPs.

Several studies [11–15] have attempted to explore the potential association of PSC and gut microbiota with 16S rRNA-based methodology using different types of sample material. A study of ileocecal biopsies using phylogenetic microarrays found that compared to ulcerative colitis (UC) and controls, colonic mucosa-associated microbiota in PSC are characterized by low diversity and a reduced abundance of uncultured Clostridiales II [11]. As for DNA sequencing-based studies, one study included biopsies from the terminal ileum, right colon and left colon, and concluded that the Barnesiellaceae family, the genus *Blautia*, and a number of OTUs (Operational Taxonomic Units) mostly representing the order Clostridiales were enriched in PSC [12]. A study of colonic tissue samples of UC patients with or without PSC found no consistent PSC-specific microbiome alterations [13]. Finally, two studies based on stool samples discovered increased abundances of different taxa when comparing healthy controls and PSC patients: the genera *Enterococcus*, *Fusobacterium*, and *Lactobacillus* in one study [14], and *Veillonella* in the other [15]. Both studies also revealed a reduced bacterial diversity in PSC.

Bile has antimicrobial properties, and the healthy biliary tract has generally been considered a sterile environment [16]. However, recent studies targeting other body sites that have also been assumed sterile, such as the lower airways and the urinary tract, have revealed microbial DNA to be present not only in pathological states, but also in healthy individuals [17, 18]. Only a few studies have used modern molecular methods to characterize the microbes present in bile, mainly in relation to various disease conditions [19–22]. Overall, biliary microbial communities appear similar to those of the upper digestive tract and have been suggested to originate from there [19, 20].

Regarding the potential association of biliary bacteria and PSC, an early culture-based study showed that bacteria, particularly streptococci, could be obtained from bile samples of patients with PSC, but not from those with primary biliary cirrhosis (PBC) [23]. They also noted that a likely source for these bacteria were previous endoscopic retrograde cholangiography (ERC) procedures. A more recent study used a 16S rRNA amplicon sequencing approach to characterize the biliary bacterial communities of 39 PSC patients [22]. Their analysis focused on microbiome changes related to patients’ genetic features, and did not include any non-PSC controls. We are not aware of any studies using modern high-throughput amplicon sequencing methodology to compare biliary microbiota of PSC patients and non-PSC subjects.

In the present study, we set out to explore the role of biliary microbiota

1. in the etiopathogenesis of PSC by comparing non-PSC controls to newly diagnosed early-stage PSC patients (ERC severity score < 6);
2. in disease progression by comparing early-stage PSC patients to advanced-stage patients (ERC severity score ≥ 6);
3. in the development of biliary dysplasia and cholangiocarcinoma (CCA) by comparing advanced-stage PSC patients to patients with dysplasia/carcinoma; and
4. the overall relationship of microbiota and disease severity as measured with the ERC score.

In addition to the PSC-related comparisons, we also studied the impact of the number of ERC examinations, since as has been noted before [23], these are likely to affect the microbiota, and repeated examinations could be an important confounding factor.

## Materials and methods

The clinical part of the study was conducted at the Helsinki University Clinic of Gastroenterology. The patients were recruited from the Clinic’s PSC registry. Informed consent was obtained from each patient, and the study was approved by the Ethics Committee of Internal Medicine in accordance to the ethical guidelines of the 1975 Declaration of Helsinki (Dnro 278/13/03/01/2009). The subject population consisted of 80 patients with PSC and 46 controls, for a total of 126 subjects. The control subjects were patients referred for their first ERC due to inconclusive bile duct MRCP findings or elevation of serum alkaline phosphatase (ALP) of unknown origin. PSC was excluded with ERC and in most cases with liver histology and clinical follow up. Detailed clinical information on the study subjects can be found in Table 1. Exclusion criteria were age under 18 years and use of antibiotics within one month before the study.

**Table 1:** Clinical and laboratory data on the study subjects.

	Controls N = 46	Early Disease N = 37	Advanced Disease N = 32	Dysplasia/Carcinoma N = 11	*p*-value
Female, n (%)	27 (59)	25 (68)	17 (50)	9 (82)	0.20
Age, years, mean (SD)	43 (15)	38 (14)	41 (13)	40 (15)	0.37
Weight, kg, mean (SD)	76 (15)	77 (15)	77 (15)	76 (15)	0.98
BMI, mean (SD)	26.3 (4.8)	25.3 (5.1)	25.2 (4.1)	26.3 (4.8)	0.67
IBD present, n (%)	9 (20)	25 (68)	20 (59)	8 (73)	<0.001
PSC duration, median (IQR)	-	0 (0, 0)	3 (, 10)	0 (0, 11)	<0.001
Age at diagnosis of PSC, mean (SD)	-	38 (13)	36 (13)	35 (14)	0.024
ERC score (0–16)	-	2.8 (1.1)	7.9 (1.8)	10.1 (2.3)	<0.001
Number of ERCs, n (%)	-				<0.001
1–2	-	34 (92)	14 (41)	4 (36)	-
3–4	-	3 (8)	8 (24)	4 (36)	-
≥5	-	0 (0)	12 (35)	3 (27)	-
HKR, % (39–50)	-	40.8 (4.1)	40.6 (3.8)	39.3 (7.3)	0.73
ESR, mm (<20)	-	19.1 (14.9)	12.2 (7.8)	22.6 (18.3)	0.095
CRP, mg/l (<3)	-	11.8 (16.8)	5.1 (4.2)	5.9 (3.8)	0.12
ALP, U/l (35–105)	-	181 (152)	192 (108)	263 (220)	0.41
AST, U/l (15–45)	-	57.8 (63.2)	69.4 (71.5)	54.6 (37.7)	0.81
ALT, U/l (<50)	-	102 (143)	96 (123)	76 (47)	0.92
Cholesterol, mmol/l	-	4.52 (1.45)	4.86 (1.20)	4.82 (1.19)	0.66
S-GT, U/l	-	220 (312)	273 (234)	335 (254)	0.58
S-bilirubin, μmol/l	-	15.7 (14.0)	12.3 (9.4)	14.6 (6.9)	0.65
B-platelets, E9/l, (150–360)	-	282 (94)	239 (75)	394 (174)	0.004
S-CEA, kU/l (0–5)	-	1.72 (0.84)	2.09 (1.51)	1.55 (0.63)	0.46
S-Ca19-9 kU/l (<26)	-	14.1 (17.3)	11.2 (12.0)	10.7 (9.9)	0.77
Biliary Lymphocytes (0–2)	-	0.88 (0.59)	0.53 (0.57)	0.70 (0.48)	0.045
Biliary Neutrophils (0–2)	-	0.68 (0.64)	0.94 (0.88)	1.00 (0.67)	0.28

The indications for ERC examination included: 1) constantly elevated ALP levels in conjunction with IBD, or 2) magnetic resonance cholangiography findings, or 3) liver biopsy suggestive of PSC, or 4) biliary dysplasia surveillance. ERC was performed using needle knife and guide wire for cannulation and the balloon catheter occlusion technique to ensure adequate filling of intra- and extrahepatic bile ducts. Before injecting contrast media, a bile sample was aspirated from the extrahepatic bile ducts with a balloon catheter. The sample was divided into 1 ml plastic tubes and immediately immersed in liquid nitrogen (-196°C). Samples were stored at -20 ^o^C until transfer to the sequencing facility, where they were stored at -80 ^o^C. In addition, brush cytology was systematically performed during ERC regardless of the presence or absence of dominant strictures. Cholangiographic findings were scored according to the modified Amsterdam score (mAm score) [24]. Both intra- and extrahepatic changes were scored, and a sum score was calculated. At the time of ERC, blood samples for routine laboratory values were collected and analysed in the laboratory of the Helsinki University Hospital (Huslab) using appropriate methods.

### Analysis of brush cytology specimens

The tip of the cytological brush was cut off, and the brush and the fluid from the brush catheter shaft were flushed into 50% ethanol. Cytospin slides were prepared and stained with Papanicolaou stain, and a cell block was also prepared when possible. Sections from cell blocks were stained with haematoxylin and eosin. The brush cytology slides were re-evaluated to reach a consensus blinded to the clinical status of the patient. Neutrophilic inflammation was evaluated semi quantitatively as described previously [25]. Brush cytology was graded as benign (including inflammatory/regenerative atypia), suspicious (suspicious for malignancy/cytological dysplasia), or malignant (CCA) using generally accepted cytological criteria.

### DNA extraction, library preparation, and sequencing

DNA extraction, amplification, and sequencing were performed at the Institute of Biotechnology, University of Helsinki. Bulk DNA was extracted from centrifuged and pelleted bile using Invisorb® Spin Blood Mini Kit (Stratec Molecular, Berlin, Germany), according to the manufacturer’s instructions. The V1-V3 regions of the bacterial 16S rRNA gene were amplified following a previously described protocol [26], except that the first PCR round included two 25μl technical replicates, and the two PCR rounds were performed with 15 and 18 cycles, respectively. The amount of template DNA for the first round ranged from 2.8 ng to 352 ng per reaction. DNA extraction kit blanks (with no template) and PCR blanks (also with no template), associated with each DNA extraction or PCR batch, were amplified and sequenced for identification of potential contaminating DNA. Finally, the PCR products were pooled together in equal concentrations and sequenced with MiSeq (Illumina, San Diego, CA) using the v3 600 cycle kit with paired-end reads (325 bp + 285 bp). Due to the amount of samples, they were split into three separate sequencing runs.

### Bioinformatics and statistical analysis

Bioinformatics and statistical analyses were performed at the Institute of Biotechnology, University of Helsinki. Primers and low quality bases and sequences were removed with CutAdapt [27]. mothur [28] was used to process the sequence data and to perform taxonomic assignments following the OTU (Operational Taxonomic Unit, a DNA sequence-based proxy for bacterial species) approach from the MiSeq Standard Operating Procedure [29, 30]. All singleton sequences were discarded to aid in processing the data as well as to reduce the number of unique sequences that are likely to be caused by sequencing errors. The raw sequence data are available at the European Nucleotide Archive with accession number PRJEB15501. Prior to any further comparisons, all OTUs of the genera *Ralstonia*, *Shewanella*, and *Halomonas* were removed as probable contaminants based on sequenced blanks, previous personal experience, as well as reported cases in the literature [31].

All statistical analyses were performed with the R programming language [32]. Double-tailed *p*-values were used throughout the study, with *p* ≤ 0.05 considered as statistically significant. Potential batch effects from the DNA isolation date and sequencing run were evaluated with Non-metric Multidimensional Scaling plots (NMDS) based on Bray-Curtis dissimilarity. The pattern for sequencing run was suggestive of batch effects, which warranted using this variable as a confounder in other comparisons.

Alpha diversity (which quantifies the “species” richness and evenness of the microbial communities) was estimated with the Shannon index, calculated with the phyloseq package [33] using non-rarefied data and compared between subject groups of interest using Kruskal-Wallis and pairwise Wilcoxon rank-sum tests.

Generalized linear models with negative binomial distribution as implemented in the DESeq2 package [34] were used to estimate differential abundances of taxa. The Benjamini-Hochberg method was used for multiple comparisons correction. Before comparisons, all taxa that were not represented by more than one sequence per sample in more than ten samples were removed. This pre-filtering was performed since rare taxa are particularly prone to produce false positives due to chance effects. This approach to low count taxa is conservative, given that DESeq2 also performs automatic filtering using the mean of normalised counts.

Three different statistical models were run in DESeq2:

1. A model in which the early stage PSC group contained only individuals referred for their first ERC examination, for comparison 1 (controls *vs* early stage PSC).
2. A model that included all subjects, with PSC status as a categorical variable, for comparisons 1 to 3 (1. controls *vs* early stage PSC; 2. early *vs* advanced stage PSC; 3. advanced stage PSC *vs* dysplasia/carcinoma).
3. A model with ERC score as a numerical descriptor of disease severity (comparison 4).

The second and third models included the number of ERC examinations as a numerical variable. All three models also included variables for IBD status and sequencing run to correct for their respective potential confounding effects.

After the DESeq2 comparisons, all statistically significant hits were further evaluated visually using box- and scatter plots of taxon relative abundance *vs* variable of interest to assess the robustness of the results. This assessment was performed to look for features suggesting that the taxa might be false positives, such as 1) putative outliers, 2) influential points with high leverage, 3) very low mean abundances, and 4) inconsistent abundance increase and decrease patterns across the groups that are difficult to explain as biologically significant.

## Results

Based on the clinical results, we classified the study subjects with PSC as follows: 37 patients were at an early disease stage (ERC severity score < 6), 32 at an advanced disease stage (ERC severity score ≥ 6), and 11 had biliary dysplasia/cholangiocarcinoma. 20% of control subjects had IBD, as opposed to 68%, 59% and 73% in the three PSC patient groups (Table 1).

**Fig 1:**
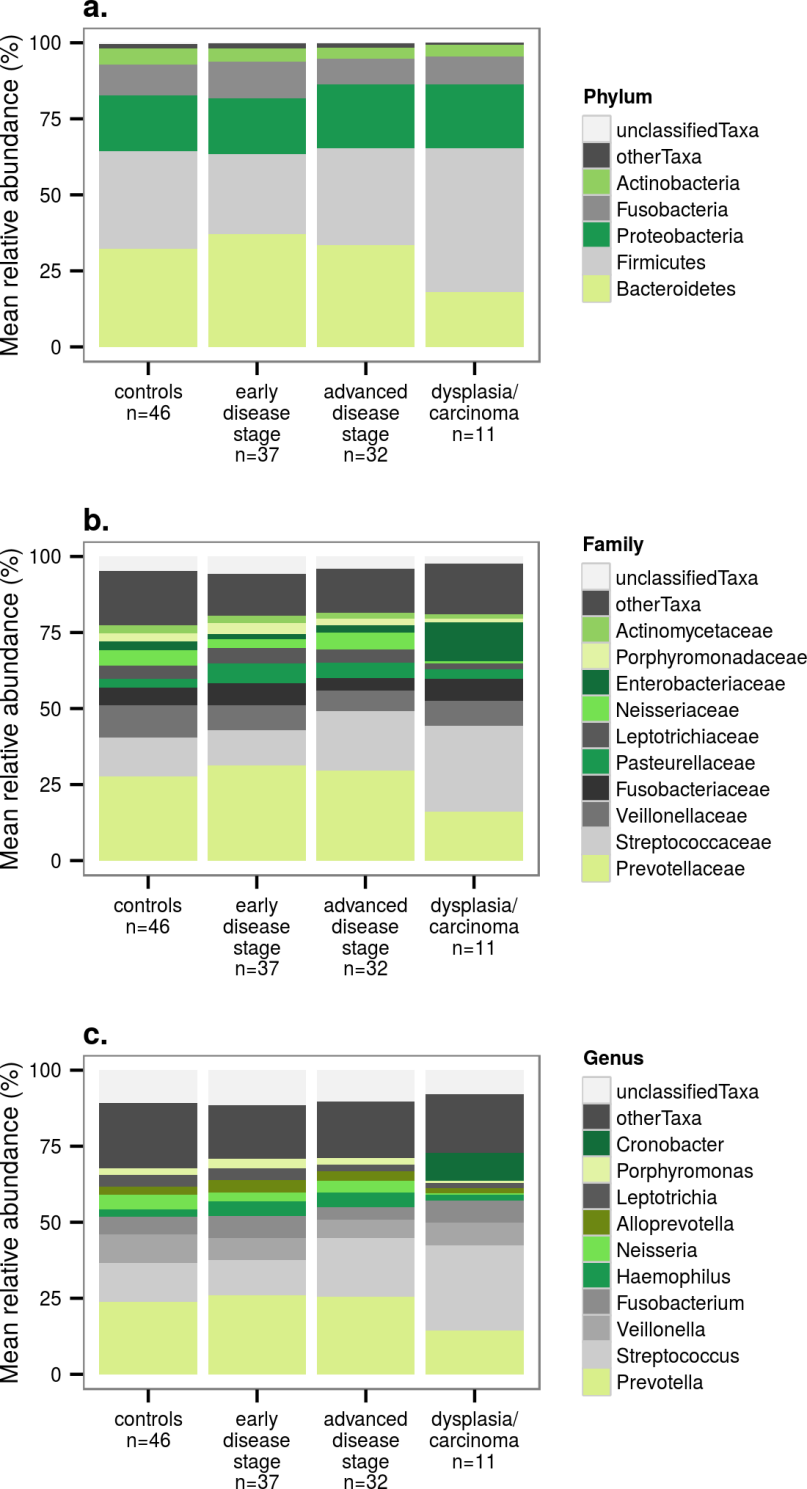
Relative abundances (%) of bacteria in controls and patients at different stages of PSC. **a)** Five most common phyla. **b)** Ten most common families. **c**) Ten most common genera.

The microbiome sequence data includes 20 bacterial phyla, subdivided into 124 families, 309 genera, and 2125 OTUs. The total number of sequences in the data set is 3 740 318, with a minimum of 2488 and a maximum of 101 556 per sample, and an average of 29 686 sequences. The most common phyla are Bacteroidetes, Firmicutes, Proteobacteria, Fusobacteria, and Actinobacteria (Fig 1A), the most common families Prevotellaceae, Streptococcaceae, Veillonellaceae, Fusobacteriaceae, and Pasteurellaceae (Fig 1B), and the most common genera *Prevotella*, *Streptococcus*, *Veillonella*, *Fusobacterium*, and *Haemophilus* (Fig 1C). The bar charts suggest that the dysplasia/carcinoma patients might have a higher abundance of bacteria of phylum Firmicutes and genus *Streptococcus* and a lower abundance of phylum Bacteroidetes and genus *Prevotella* than the other groups, and that the abundance of *Streptococcus* could also be higher in advanced stage PSC patients than in controls or early stage patients.

### Microbial diversity

**Fig 2:**
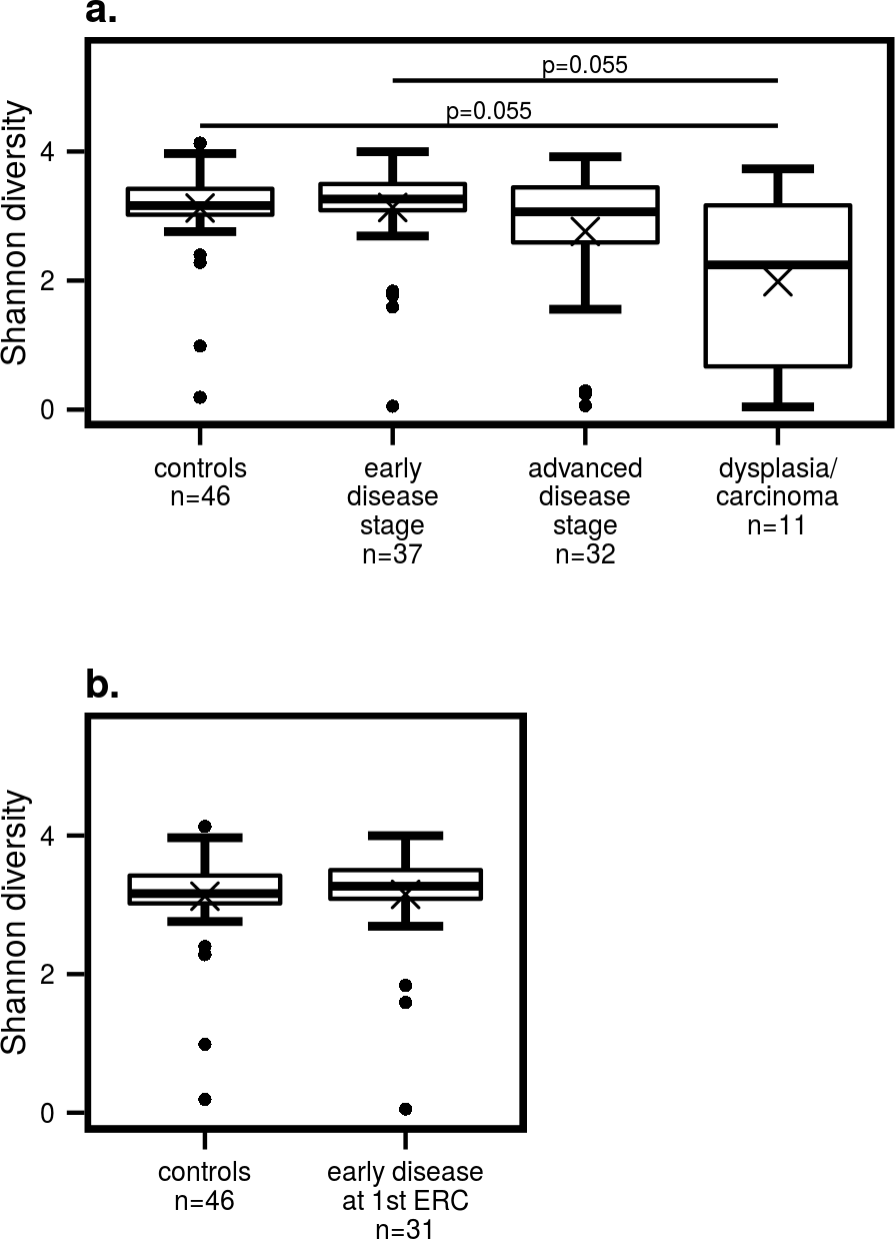
Shannon diversity. **a)** in controls and patients at different stages of PSC. **b)** in controls compared to early stage patients at their 1^st^ ERC examination. Lower and upper box hinges represent the 1^st^ and 3^rd^ quartiles, respectively. Whiskers represent 1.5 times the interquartile range. Bold lines represent medians and crosses the means.

Comparisons of microbial alpha diversity, estimated with the Shannon index, suggest that there are differences between disease stages (*p* = 0.036, Kruskal-Wallis rank sum test). Further, pairwise comparison of groups does not show statistically significant differences between any two groups (*p* ≥ 0.055, pairwise Wilcoxon rank sum test), but the low *p*-values nevertheless suggest that differences are present and are more pronounced between controls and early disease patients against the dysplasia/carcinoma stage, with the advanced disease stage falling between the two (Fig 2A). When the microbiota of control subjects are compared to that of PSC patients with early stage at their first ERC examination (to exclude any effects of the procedure), no statistically significant differences are detected (*p* = 0.64, pairwise Wilcoxon rank sum test; Fig 2B). In general, visual inspection of the groups’ diversity progression pattern and the above test results suggest that diversity is similar between controls and the early disease stage, and then drops progressively through the advanced and dysplasia/carcinoma stages.

### Microbial differential abundance

Differential abundance assessment using DESeq2 results in a long list of potential taxa of interest in all group comparisons (S1 Table). Comparing the control patients and the early stage PSC patients at their first ERC examination reveals four OTUs (an unclassified Enterobacteriaceae OTU, Otu0008; *Neisseria* Otu00045; *Campylobacter* Otu00089; and an unclassified Neisseriaceae OTU, Otu00213) and three families (Pasteurellaceae, Staphylococcaceae, and Xanthomonadaceae) as differentially abundant, with no genus-level results (Table 2, S1 Fig). When contrasting the control group with the early disease group as a whole, we obtain statistical significance for two OTUs (an unclassified Clostridiales, Otu00188, and the same unclassified Neisseriaceae as above, Otu00213) and one family, Staphylococcaceae (Table 2, S2 Fig). However, a visual assessment of these taxa (S1 and S2 Figs) suggests that many of these taxa might be false positives.

**Table 2:** Statistically significant results from differential abundance comparisons.

Family	Genus	OTU	Mean Abundance	Log 2 Fold Change	Standard Error of Log 2 Fold Change	p-value	Adjusted p-value
**Controls -> patients with early disease and no history of ERC examinations**							
Enterobacteriaceae	unclassified	Otu00008	640	-4,65	1,27	2,42E-04	1,98E-02
Neisseriaceae	Neisseria	Otu00045	33	-3,18	0,94	7,14E-04	3,78E-02
Campylobacteraceae	Campylobacter	Otu00089	11	-2,79	0,77	2,80E-04	1,98E-02
Neisseriaceae	unclassified	Otu00213	1	-5,01	1,17	1,78E-05	3,78E-03
Pasteurellaceae	-	-	14058	1,95	0,64	2,12E-03	3,25E-02
Staphylococcaceae	-	-	179	3,97	0,76	1,73E-07	7,95E-06
Xanthomonadaceae	-	-	11	3,58	1,11	1,20E-03	2,76E-02
**Controls -> patients with early disease**							
unclassified Clostridiales	unclassified	Otu00188	1	-6,10	1,41	1,43E-05	1,52E-03
Neisseriaceae	unclassified	Otu00213	1	-4.91	1.10	7.64E-06	1.52E-03
Staphylococcaceae	-	-	179	4,18	0,76	4,09E-08	2,25E-06
**Patients with early disease -> patients with advanced disease**							
Streptococcaceae	Streptococcus	Otu00020	728	4.94	0.83	3.22E-09	3.93E-07
Streptococcaceae	Streptococcus	-	9241	1.44	0.42	6.70E-04	4.94E-03
**Patients with advanced disease -> patients with dysplasia/carcinoma**							
Streptococcaceae	Streptococcus	Otu00035	602	5.78	1.31	1.00E-05	7.10E-04
Streptococcaceae	Streptococcus	Otu00061	23	4.02	1.09	2.34E-04	8.25E-03
Prevotellaceae	Prevotella	Otu00128	6	-4.41	1.40	1.63E-03	3.15E-02
**ERC severity score as a numerical variable**							
Streptococcaceae	Streptococcus	Otu00020	728	0.28	0.09	1.08E-03	4.35E-02
Streptococcaceae	Streptococcus	Otu00061	23	0.35	0.08	2.14E-05	1.74E-03
**Number of ERC examinations (statistical model with disease severity as a grouped factor variable)**							
Streptococcaceae	Streptococcus	Otu00035	602	1.41	0.22	1.11E-10	2.54E-08
Streptococcaceae	Streptococcus	-	9241	0.39	0.10	1.25E-04	2.63E-03
**Number of ERC examinations (statistical model with ERC severity score as a numerical variable)**							
Streptococcaceae	Streptococcus	Otu00035	606	0.94	0.23	5.43E-05	1.08E-03
Streptococcaceae	Streptococcus	-	9241	0.46	0.11	1.18E-05	4.96E-04

**Legend.** Statistically significant results from differential abundance comparisons. The two contrasts between the control group and the early disease groups contain all the original significant results, for the reader’s convenience. The next five comparisons contain only the results that passed assessment of robustness. A full table containing all statistically significant results from all models and contrasts of interest can be found in the Supplement (S1 Table). **Mean abundance** = mean taxon abundance (number of sequences) across data set after normalization for sequencing depth.

**Fig 3:**
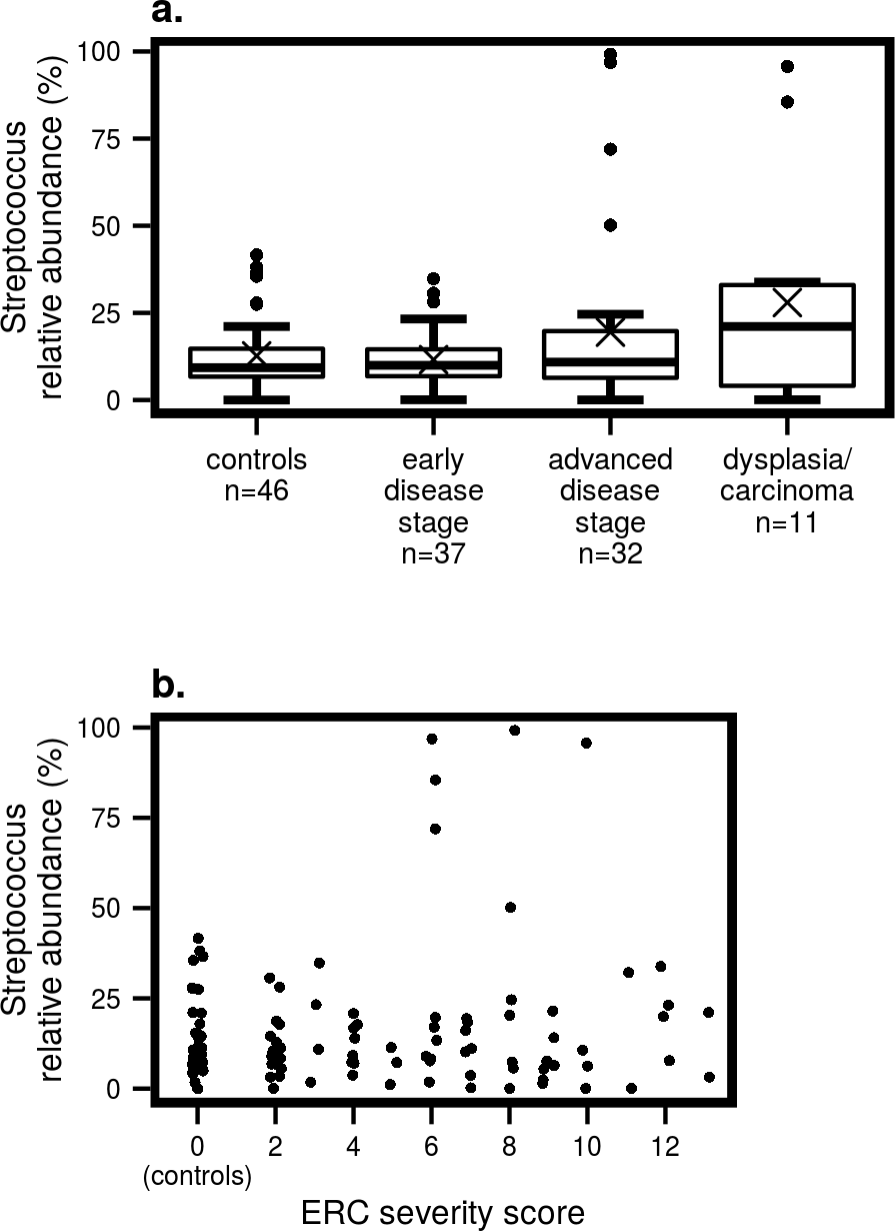
Relative abundances (%) of the genus *Streptococcus*. **a)** in controls and patients at different stages of PSC. **b)** in relation to ERC score. Lower and upper box hinges represent the 1^st^ and 3^rd^ quartiles, respectively. Whiskers represent 1.5 times the interquartile range. Bold lines represent medians and crosses the means.

For the other comparisons, we find a total of 24 taxa with a statistically significant difference in abundance between early and advanced stage patients, and 36 when contrasting advanced stage patients and patients with dysplasia or carcinoma (S1 Table). After exploring these taxa visually, we consider the streptococcal group the most important one, showing a consistent, robust pattern coupled to meaningful mean abundances (Table 2, S1 Table). Our comparisons indicate that there is a statistically significant difference between early and advanced stage patients in the abundances of genus *Streptococcus* (Fig 3A) and one *Streptococcus* OTU (Otu00020), and between advanced disease patients and patients with dysplasia/carcinoma in two *Streptococcus* OTUs (Otu00035 and Otu00061). Two *Streptococcus* OTUs (Otu00020 and Otu00061) are also significant when disease severity is measured using the ERC score as a numerical variable (Fig 3B). Finally, a low abundance *Prevotella* OTU (Otu00128) is reduced to zero abundance in the dysplasia/carcinoma group.

Since previous ERC examinations could have had an effect on the biliary microbiome, we looked at what taxa appear to be associated with the number of ERC examinations undergone by each patient. The resulting taxa are the same for both the model where PSC patients are categorized according to severity, and the one where a numerical ERC score is used instead, suggesting that the abundances of the genus *Streptococcus* and *Streptococcus* Otu00035 increase with additional ERC examinations (Table 2).

Finally, we took the opportunity to assess the impact of IBD on bile bacterial communities in PSC. Our data does not support alpha diversity changes for IBD versus no IBD when using both controls and PSC subjects, nor among PSC subjects only (*p* = 0.9 and *p* = 0.24, respectively). Differential abundance analysis produces some statistically significant hits (S1 Table), but none of them hold as robust under close scrutiny: they seem likely to be either contaminants, misclassified (e.g. *Soonwooa*, a marine bacterium not expected to be present in human samples), or false positives affected by outliers or chance effects due to low mean abundances.

## Discussion

Microorganisms have been suggested to be involved in the etiopathogenesis of PSC, but so far, there have only been only few studies exploring the topic with modern molecular methods [7]. To our knowledge, the present study is the first one to use high-throughput 16S rRNA gene sequencing of bile material from both PSC patients and non-PSC control subjects to assess the role of microbiota in the initiation, progression and development of dysplasia in PSC. Our study subjects included patients with newly diagnosed PSC at their first ERC examination, patients at an early disease stage but with a history of multiple ERC examinations, patients with advanced biliary disease, and those presenting with biliary neoplasia. We found evidence for a decrease in bacterial diversity in patients with dysplasia or cholangiocarcinoma. Looking at specific bacterial taxa, the genus *Streptococcus* appears to be of particular interest, with the genus itself and several OTUs being differentially abundant between the groupings under comparison.

### Bile microbiota and the etiopathogenesis of PSC

Our results do not support a diversity difference in microbiota between controls and PSC patients with early-stage. None of the taxa reported as differentially abundant (except for Neisseriaceae Otu00213) show convincing, robust patterns under visual inspection, and they could very well be false positives, although it would be premature to disregard them. Otu0008, Otu00045, Otu00089, Otu00213, and Otu00188 are all less abundant in the early stage PSC group than in controls, which argues against an etiological role in PSC. Also, except for Otu0008, all show extremely low mean abundances. The families Pasteurellaceae, Staphylococcaceae, and Xanthomonadaceae all contain known human pathogens, but all are quite diverse as a group. Given that no specific genera or OTUs representing these families are differentially abundant, and Xanthomonadaceae has a very low mean abundance, the results do not suggest an infectious role in the initiation of PSC. Overall, our data doesn’t provide convincing evidence that bile microbiota are involved in the initiation of bile duct inflammation or in the aetiology of PSC. Finally, it should be noted that our controls’ microbial communities may not represent “normal” or fully healthy bile microbiota, as they all had some indication for undergoing an ERC.

### Role of bile microbiota in disease progression

Our results indicate that microbial diversity could be reduced between early and advanced stage patients. This could be thought to mirror the pattern seen in several studies of gut-related microbial communities where PSC subjects were found to have a reduced diversity compared to controls [11, 14, 15]. There are significant differences in the abundances of streptococci between early and advanced stage patients, both for a specific OTU and the entire genus (Fig 3A, Table 2). Two OTUs are also positively correlated with disease severity as measured with the ERC score (Fig 3B). Based on these findings, streptococci could have a role in disease progression, even if they might not be involved in the initiation of PSC. Both the genus *Streptococcus* and one specific OTU are also positively correlated with the number of ERC examinations performed (Table 2). A previous study concluded that the streptococci cultured from bile samples of PSC patients are primarily a consequence of ERCs [23]. However, our statistical model takes into account both disease severity and the history of multiple ERC examinations, and streptococci appear associated with both. Bacteria of the genus *Streptococcus* are also detectable in bile samples of control subjects and early-stage PSC patients with no history of previous ERC examinations, suggesting that the genus might be constantly present in bile, although it is not possible to completely exclude contamination during sample collection. Therefore, the potential role of streptococci in disease progression calls for further attention, while their association with the number of ERCs underlines the risk of nosocomial infection during the procedure.

### Bile microbiota and development of biliary neoplasia

Our results showed that biliary microbial diversity is the lowest in patients with dysplasia or cholangiocarcinoma (Fig 2A). As for differential abundance analyses, two *Streptococcus* OTUs appear more abundant in dysplasia/carcinoma than in advanced stage PSC patients (Table 2). The genus *Streptococcus* is also more abundant in dysplasia/carcinoma patients’ bile, although this difference is not statistically significant (Fig 3A). The differences in diversity and *Streptococcus* abundance seem to follow the same trend as those seen in the comparison between early and late stage patients, indicating a progressive reduction in bacterial diversity and a generalised increase in streptococcal abundance as PSC develops.

### Limitations of the study

This study was designed to be as representative as possible of the main disease stages within the limitations of a sample of convenience, i.e. to have an adequate number of samples representative of the groups under study within the practical restrictions of obtaining suitable bile samples. We are primarily looking for pathogenic organisms associated with the aetiology and/or development of PSC, and in the case of an infection we would expect that any candidate organism(s) would be clearly overrepresented in PSC. The sample sizes in our study should be adequate to constrain interpersonal variation of microbiota for this particular purpose, with the possible exception of the dysplasia/carcinoma group. Additionally, the analysis with the ERC severity score uses a numerical scale and all 126 samples, and in this case we do not think that sample size would limit the detection of potential pathogens in a clear clinical infection scenario. On the other hand, we are dealing with a complex disease in which more subtle microbiome effects could be at play. Thus, we can’t exclude the possibility of substantially more intricate relationships between bile microbial communities and PSC. Larger sample sizes could have allowed the detection of more subtle differences in bacterial abundances that could still have biological meaning in the context of the disease.

## Conclusions

The results of our exploratory study suggest that the aetiology of PSC is not associated with specific changes in biliary microbial communities. However, members of the *Streptococcus* genus appear to be positively correlated with disease progression, even when the number of previous ERC examinations is controlled for. ERC examinations are also associated with an increase of streptococcal abundance, supporting previous findings that the procedure might exacerbate the growth of these bacteria. Also of interest are the findings regarding alpha diversity, which is estimated to decrease gradually from the early disease stage to biliary neoplasia, even though the diversity of naïve PSC patients is similar to that of non-PSC controls. It is not possible to evaluate based on our results whether these changes in the microbiome are directly associated with the disease process, or a product of biliary bacterial communities adjusting to the changes in their environment. Either way, our study underlines the need to further explore the role of *Streptococcus* in PSC. Studies of what constitutes a “normal” biliary microbiome, if there indeed is one, would be crucial for better understanding the disease-related changes.

## Supporting information

S1 Fig Families and OTUs from differential abundance analysis between the control group and early stage PSC patients at their first ERC examination. Whiskers represent 1.5 times the interquartile range. Bold lines represent medians and crosses the means.
(PDF)

S2 Fig Families and OTUs from differential abundance analysis between the control group and the early disease group as a whole. Whiskers represent 1.5 times the interquartile range. Bold lines represent medians and crosses the means.
(PDF)

S1 Table Complete list of statistically significant results. All statistically significant results from all the GLMs used in this study for differential abundance analysis, except for those associated with the sequencing run variable, which was used only for controlling purposes. **Mean abundance** = mean taxon abundance (number of sequences) across data set after normalization for sequencing depth.
(PDF)

## References

[1] Hirschfield GM, Karlsen TH, Lindor KD, Adams DH. Primary sclerosing cholangitis. Lancet. 2013;382(9904):1587–99. doi: 10.1016/S0140-6736(13)60096-3 23810223 10.1016/S0140-6736(13)60096-3

[2] Bergquist A, Ekbom A, Olsson R, Kornfeldt D, Loof L, Danielsson A, et al Hepatic and extrahepatic malignancies in primary sclerosing cholangitis. Journal of hepatology. 2002;36(3):321–7. 11867174 10.1016/s0168-8278(01)00288-4

[3] Karlsen TH, Boberg KM. Update on primary sclerosing cholangitis. Journal of hepatology. 2013;59(3):571–82. doi: 10.1016/j.jhep.2013.03.015 23603668 10.1016/j.jhep.2013.03.015

[4] Tabibian JH, O'Hara SP, Lindor KD. Primary sclerosing cholangitis and the microbiota: current knowledge and perspectives on etiopathogenesis and emerging therapies. Scand J Gastroenterol. 2014;49(8):901–8. doi: 10.3109/00365521.2014.913189 24990660 10.3109/00365521.2014.913189 PMC4210190

[5] Tabibian JH, Talwalkar JA, Lindor KD. Role of the microbiota and antibiotics in primary sclerosing cholangitis. Biomed Res Int. 2013;2013:389537 doi: 10.1155/2013/389537 24232746 10.1155/2013/389537 PMC3819830

[6] Eksteen B. Advances and controversies in the pathogenesis and management of primary sclerosing cholangitis. Br Med Bull. 2014;110(1):89–98. doi: 10.1093/bmb/ldu008 24795363 10.1093/bmb/ldu008

[7] Mattner J. Impact of Microbes on the Pathogenesis of Primary Biliary Cirrhosis (PBC) and Primary Sclerosing Cholangitis (PSC). Int J Mol Sci. 2016;17(11). 10.3390/ijms17111864 PMC5133864 27834858

[8] DuPont AW, DuPont HL. The intestinal microbiota and chronic disorders of the gut. Nat Rev Gastroenterol Hepatol. 2011;8(9):523–31. doi: 10.1038/nrgastro.2011.133 21844910 10.1038/nrgastro.2011.133

[9] Duboc H, Rajca S, Rainteau D, Benarous D, Maubert MA, Quervain E, et al Connecting dysbiosis, bile-acid dysmetabolism and gut inflammation in inflammatory bowel diseases. Gut. 2013;62(4):531–9. doi: 10.1136/gutjnl-2012-302578 22993202 10.1136/gutjnl-2012-302578

[10] Clemente JC, Ursell LK, Parfrey LW, Knight R. The impact of the gut microbiota on human health: an integrative view. Cell. 2012;148(6):1258–70. doi: 10.1016/j.cell.2012.01.035 22424233 10.1016/j.cell.2012.01.035 PMC5050011

[11] Rossen NG, Fuentes S, Boonstra K, D'Haens GR, Heilig HG, Zoetendal EG, et al The mucosa-associated microbiota of PSC patients is characterized by low diversity and low abundance of uncultured Clostridiales II. Journal of Crohn's & colitis. 2015;9(4):342–8. 10.1093/ecco-jcc/jju023 25547975

[12] Torres J, Bao X, Goel A, Colombel JF, Pekow J, Jabri B, et al The features of mucosa-associated microbiota in primary sclerosing cholangitis. Aliment Pharmacol Ther. 2016;43(7):790–801. doi: 10.1111/apt.13552 26857969 10.1111/apt.13552 PMC5177987

[13] Kevans D, Tyler AD, Holm K, Jorgensen KK, Vatn MH, Karlsen TH, et al Characterization of Intestinal Microbiota in Ulcerative Colitis Patients with and without Primary Sclerosing Cholangitis. Journal of Crohn's & colitis. 2016;10(3):330–7. 10.1093/ecco-jcc/jjv204 PMC4957469 26526357

[14] Sabino J, Vieira-Silva S, Machiels K, Joossens M, Falony G, Ballet V, et al Primary sclerosing cholangitis is characterised by intestinal dysbiosis independent from IBD. Gut. 2016;65(10):1681–9. doi: 10.1136/gutjnl-2015-311004 27207975 10.1136/gutjnl-2015-311004 PMC5036217

[15] Kummen M, Holm K, Anmarkrud JA, Nygard S, Vesterhus M, Hoivik ML, et al The gut microbial profile in patients with primary sclerosing cholangitis is distinct from patients with ulcerative colitis without biliary disease and healthy controls. Gut. 2016. 10.1136/gutjnl-2015-310500 26887816

[16] Verdier J, Luedde T, Sellge G. Biliary Mucosal Barrier and Microbiome. Viszeralmedizin. 2015;31(3):156–61. doi: 10.1159/000431071 26468308 10.1159/000431071 PMC4569210

[17] Aho VT, Pereira PA, Haahtela T, Pawankar R, Auvinen P, Koskinen K. The microbiome of the human lower airways: a next generation sequencing perspective. World Allergy Organ J. 2015;8(1):23 doi: 10.1186/s40413-015-0074-z 26140078 10.1186/s40413-015-0074-z PMC4468963

[18] Whiteside SA, Razvi H, Dave S, Reid G, Burton JP. The microbiome of the urinary tract—a role beyond infection. Nat Rev Urol. 2015;12(2):81–90. doi: 10.1038/nrurol.2014.361 25600098 10.1038/nrurol.2014.361

[19] Shen H, Ye F, Xie L, Yang J, Li Z, Xu P, et al Metagenomic sequencing of bile from gallstone patients to identify different microbial community patterns and novel biliary bacteria. Sci Rep. 2015;5:17450 doi: 10.1038/srep17450 26625708 10.1038/srep17450 PMC4667190

[20] Ye F, Shen H, Li Z, Meng F, Li L, Yang J, et al Influence of the Biliary System on Biliary Bacteria Revealed by Bacterial Communities of the Human Biliary and Upper Digestive Tracts. PLoS One. 2016;11(3):e0150519 doi: 10.1371/journal.pone.0150519 26930491 10.1371/journal.pone.0150519 PMC4773253

[21] Hiramatsu K, Harada K, Tsuneyama K, Sasaki M, Fujita S, Hashimoto T, et al Amplification and sequence analysis of partial bacterial 16S ribosomal RNA gene in gallbladder bile from patients with primary biliary cirrhosis. Journal of hepatology. 2000;33(1):9–18. 10905580 10.1016/s0168-8278(00)80153-1

[22] Folseraas T, Melum E, Rausch P, Juran BD, Ellinghaus E, Shiryaev A, et al Extended analysis of a genome-wide association study in primary sclerosing cholangitis detects multiple novel risk loci. Journal of hepatology. 2012;57(2):366–75. doi: 10.1016/j.jhep.2012.03.031 22521342 10.1016/j.jhep.2012.03.031 PMC3399030

[23] Olsson R, Bjornsson E, Backman L, Friman S, Hockerstedt K, Kaijser B, et al Bile duct bacterial isolates in primary sclerosing cholangitis: a study of explanted livers. Journal of hepatology. 1998;28(3):426–32. 9551680 10.1016/s0168-8278(98)80316-4

[24] Boyd S, Tenca A, Jokelainen K, Mustonen H, Krogerus L, Arola J, et al Screening primary sclerosing cholangitis and biliary dysplasia with endoscopic retrograde cholangiography and brush cytology: risk factors for biliary neoplasia. Endoscopy. 2016;48(5):432–9. doi: 10.1055/s-0041-110792 26808393 10.1055/s-0041-110792

[25] Boyd S, Mustonen H, Tenca A, Jokelainen K, Arola J, Farkkila MA. Surveillance of primary sclerosing cholangitis with ERC and brush cytology: risk factors for cholangiocarcinoma. Scand J Gastroenterol. 2017;52(2):242–9. doi: 10.1080/00365521.2016.1250281 27806633 10.1080/00365521.2016.1250281

[26] Salava A, Aho V, Lybeck E, Pereira P, Paulin L, Nupponen I, et al Loss of cutaneous microbial diversity during first three weeks of life in very low birth weight infants. Exp Dermatol. 2017. 10.1111/exd.13312 28156021

[27] Martin M. Cutadapt removes adapter sequences from high-throughput sequencing reads. EMBnet.journal. 2011;17(1).

[28] Schloss PD, Westcott SL, Ryabin T, Hall JR, Hartmann M, Hollister EB, et al Introducing mothur: open-source, platform-independent, community-supported software for describing and comparing microbial communities. Appl Environ Microbiol. 2009;75(23):7537–41. doi: 10.1128/AEM.01541-09 19801464 10.1128/AEM.01541-09 PMC2786419

[29] Kozich JJ, Westcott SL, Baxter NT, Highlander SK, Schloss PD. Development of a dual-index sequencing strategy and curation pipeline for analyzing amplicon sequence data on the MiSeq Illumina sequencing platform. Appl Environ Microbiol. 2013;79(17):5112–20. doi: 10.1128/AEM.01043-13 23793624 10.1128/AEM.01043-13 PMC3753973

[30] Schloss PD. Standard Operating Procedure for MiSeq sequenced 16S rRNA gene data [Available from: http://www.mothur.org/wiki/MiSeq_SOP.]

[31] Salter SJ, Cox MJ, Turek EM, Calus ST, Cookson WO, Moffatt MF, et al Reagent and laboratory contamination can critically impact sequence-based microbiome analyses. BMC biology. 2014;12:87 doi: 10.1186/s12915-014-0087-z 25387460 10.1186/s12915-014-0087-z PMC4228153

[32] R Core Team. R: A language and environment for statistical computing R Foundation for Statistical Computing, Vienna, Austria 2015 [Available from: https://www.r-project.org/.]

[33] McMurdie PJ, Holmes S. phyloseq: an R package for reproducible interactive analysis and graphics of microbiome census data. PLoS One. 2013;8(4):e61217 doi: 10.1371/journal.pone.0061217 23630581 10.1371/journal.pone.0061217 PMC3632530

[34] Love MI, Huber W, Anders S. Moderated estimation of fold change and dispersion for RNA-seq data with DESeq2. Genome Biol. 2014;15(12):550 doi: 10.1186/s13059-014-0550-8 25516281 10.1186/s13059-014-0550-8 PMC4302049

