# Supplementary figures and images for: Bile microbiota in primary sclerosing cholangitis: Impact on disease progression and development of biliary dysplasia
Source: PLoS One. 2017 Aug 10;12(8):e0182924. doi: 10.1371/journal.pone.0182924 (PMC5552186; doi:10.1371/journal.pone.0182924)

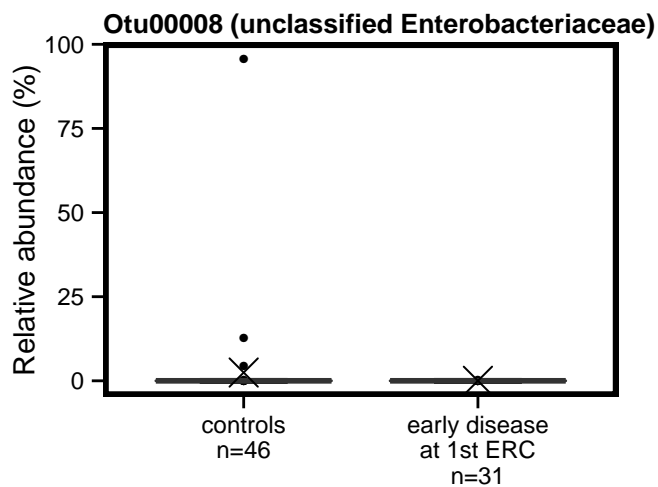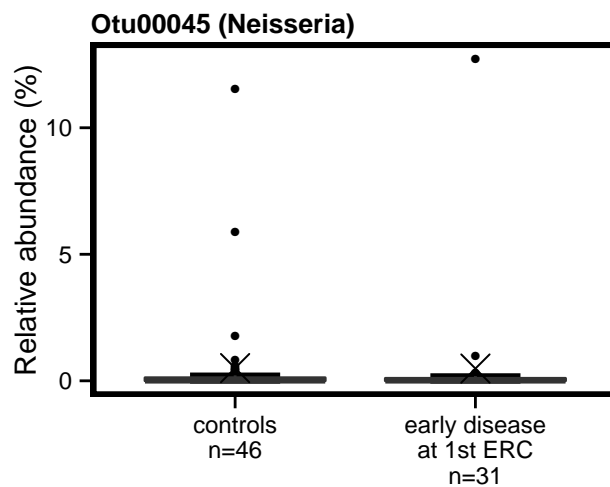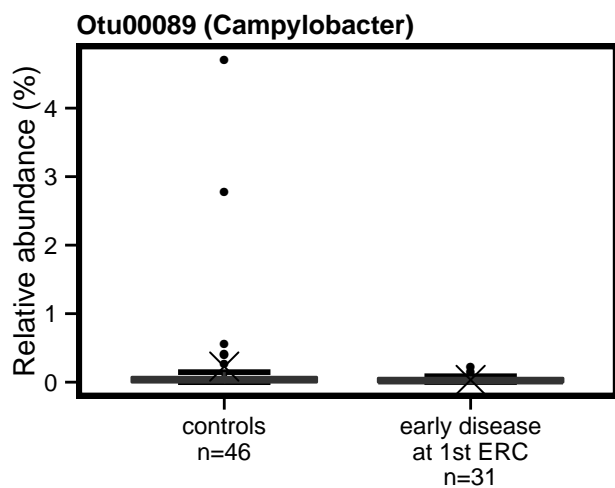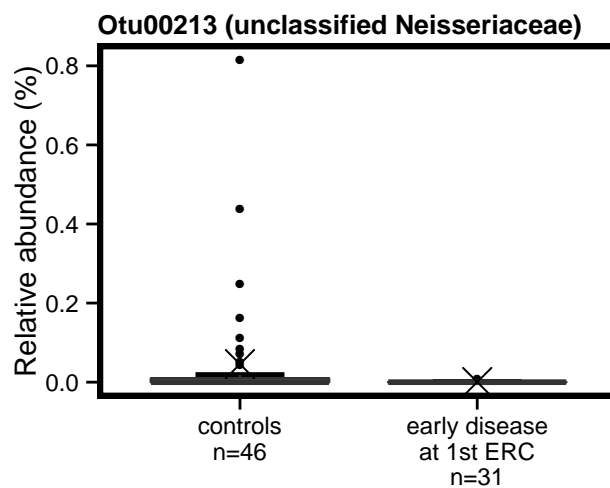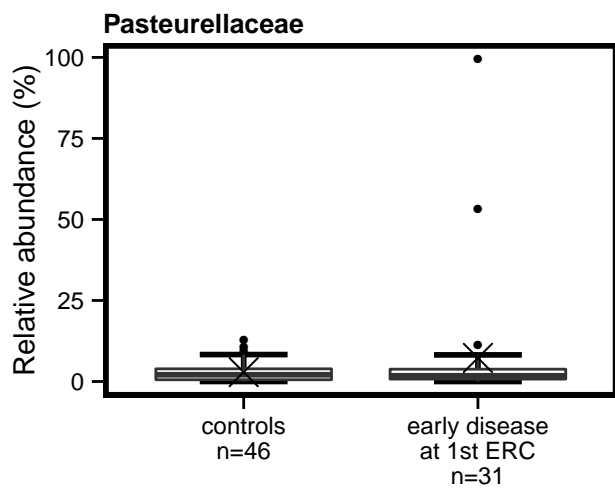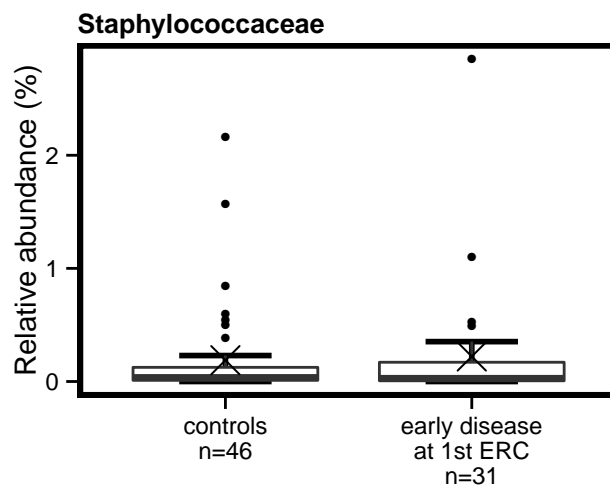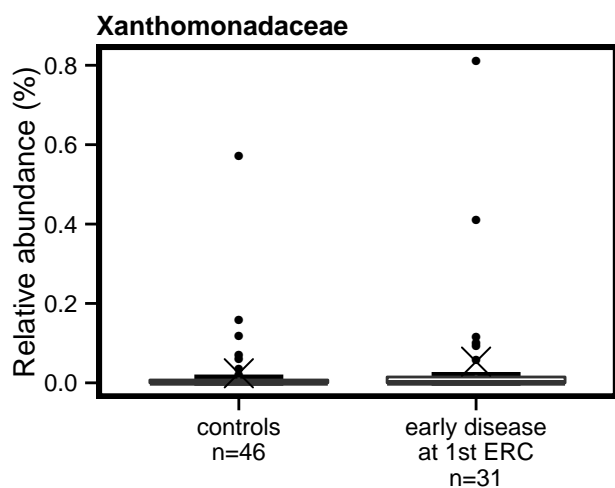

Supplement: S1 Fig — Whiskers represent 1.5 times the interquartile range. Bold lines represent medians and crosses the means. (PDF) [file pone.0182924.s001.pdf]

**Otu00188 (unclassified Clostridiales)**

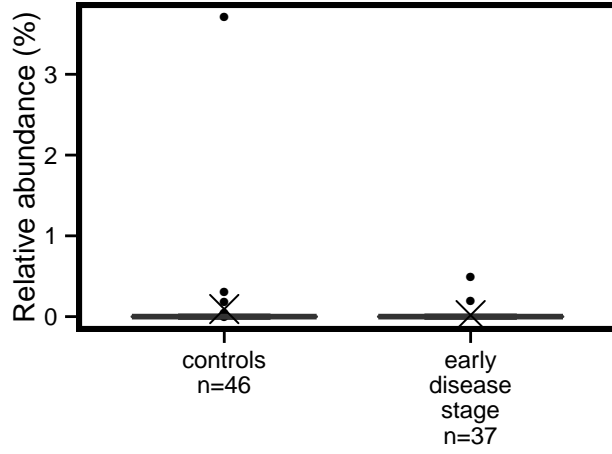

**Otu00213 (unclassified Neisseriaceae)**

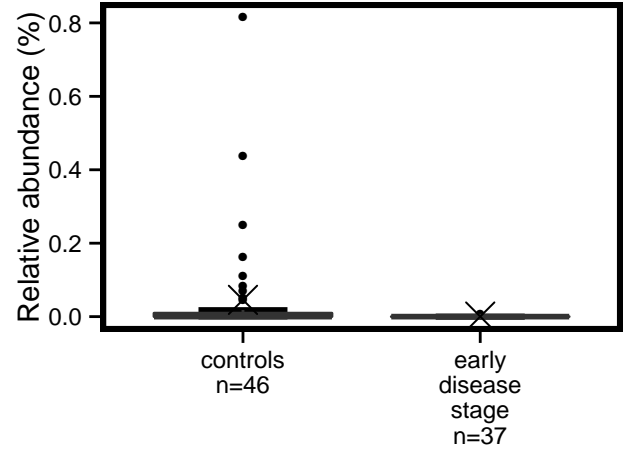

**Staphylococcaceae**

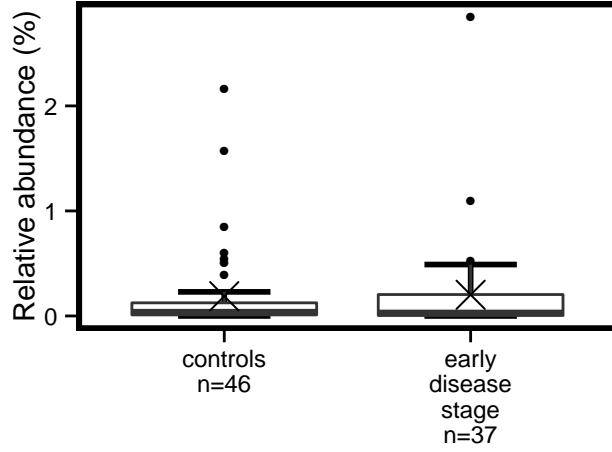

Supplement: S2 Fig — Whiskers represent 1.5 times the interquartile range. Bold lines represent medians and crosses the means. (PDF) [file pone.0182924.s002.pdf]
