# Supplementary material for: Bile microbiota in primary sclerosing cholangitis: Impact on disease progression and development of biliary dysplasia
Source: PLoS One. 2017 Aug 10;12(8):e0182924. doi: 10.1371/journal.pone.0182924 (PMC5552186; doi:10.1371/journal.pone.0182924)
Supplement: S1 Table — All statistically significant results from all the GLMs used in this study for differential abundance analysis, except for those associated with the sequencing run variable, which was used only for controlling purposes. Mean abundance = mean taxon abundance (number of sequences) across data set after normalization for sequencing depth. (PDF) [file pone.0182924.s003.pdf]

Supplement

Family	Genus	OTU	Mean Abundance	Log 2 Fold Change	Standard Error of Log 2 Fold Change	p-value	Adjusted p-value
**Model 1 (Run + IBD + Disease stage stratified by number of ERC examinations)**							
**controls -> patients with early disease and no history of ERC examinations**							
Enterobacteriaceae	unclassified	Otu00008	640	-4,65	1,27	2,42E-04	1,98E-02
Neisseriaceae	Neisseria	Otu00045	33	-3,18	0,94	7,14E-04	3,78E-02
Campylobacteraceae	Campylobacter	Otu00089	11	-2,79	0,77	2,80E-04	1,98E-02
Neisseriaceae	unclassified	Otu00213	1	-5,01	1,17	1,78E-05	3,78E-03
Pasteurellaceae	-	-	14058	1,95	0,64	2,12E-03	3,25E-02
Staphylococcaceae	-	-	179	3,97	0,76	1,73E-07	7,95E-06
Xanthomonadaceae	-	-	11	3,58	1,11	1,20E-03	2,76E-02
**Model 2 (Run + IBD + number of ERC examinations + Disease stage)**							
**controls -> patients with early disease**							
unclassified Clostridiales	unclassified	Otu00188	1	-6,10	1,41	1,43E-05	1,52E-03
Neisseriaceae	unclassified	Otu00213	1	-4,91	1,10	7,64E-06	1,52E-03
Staphylococcaceae	-	-	179	4,18	0,76	4,09E-08	2,25E-06
**patients with early disease -> patients with advanced disease**							
Pasteurellaceae	Haemophilus	Otu00005	2504	3,44	0,69	6,35E-07	5,16E-05
Streptococcaceae	Streptococcus	Otu00020	728	4,94	0,83	3,22E-09	3,93E-07
Propionibacteriaceae	Propionibacterium	Otu00024	227	2,74	0,63	1,42E-05	8,67E-04
Bradyrhizobiaceae	Bradyrhizobium	Otu00031	530	4,22	0,64	3,38E-11	8,25E-09
Carnobacteriaceae	Granulicatella	Otu00108	48	4,16	1,01	3,94E-05	1,92E-03
Carnobacteriaceae	Granulicatella	Otu00392	6	-3,23	0,84	1,23E-04	5,01E-03
Bradyrhizobiaceae	Bradyrhizobium	-	4916	5,56	0,70	2,26E-15	1,33E-13
Burkholderiaceae	Burkholderia	-	1766	10,53	1,74	1,30E-09	2,55E-08

Corynebacteriaceae	Corynebacterium	-	221	2,46	0,61	4,95 E-05	4,87E-04
Carnobacteriaceae	Granulicatella	-	10957	1,89	0,61	1,88 E-03	1,23E-02
Pasteurellaceae	Haemophilus	-	39637	5,19	0,73	1,37 E-12	4,05E-11
Streptococcaceae	Lactococcus	-	19	-4,44	1,68	8,20 E-03	4,84E-02
Propionibacteriaceae	Propionibacterium	-	1883	4,18	0,70	1,95 E-09	2,87E-08
Flavobacteriaceae	Soonwooa	-	7	5,76	1,69	6,47 E-04	4,94E-03
Streptococcaceae	Streptococcus	-	9241	1,44	0,42	6,70 E-04	4,94E-03
Bradyrhizobiaceae	-	-	7672	4,79	0,77	4,82 E-10	9,88E-09
Burkholderiaceae	-	-	2716	10,71	1,61	2,73 E-11	1,12E-09
Carnobacteriaceae	-	-	2430	2,26	0,62	2,53 E-04	1,48E-03
Comamonadaceae	-	-	2011	3,87	1,11	4,91 E-04	2,52E-03
Corynebacteriaceae	-	-	302	3,33	0,64	2,24 E-07	1,83E-06
Lachnospiraceae	-	-	704	1,73	0,53	1,15 E-03	5,25E-03
Pasteurellaceae	-	-	13860	3,87	0,69	2,09 E-08	2,86E-07
Propionibacteriaceae	-	-	2913	3,47	0,73	1,91 E-06	1,31E-05
Ruminococcaceae	-	-	347	8,22	1,57	1,63 E-07	1,67E-06
**patients with advanced disease -> patients with dysplasia/carcinoma**							
Pasteurellaceae	Haemophilus	Otu00 005	2504	-3,68	0,99	1,93 E-04	8,18E-03
Neisseriaceae	Neisseria	Otu00 006	272	-4,07	1,02	6,82 E-05	3,61E-03
Fusobacteriaceae	Fusobacterium	Otu00 011	290	-2,65	0,89	3,02 E-03	4,49E-02
Carnobacteriaceae	Granulicatella	Otu00 018	1650	4,42	0,72	7,14 E-10	1,51E-07
Streptococcaceae	Streptococcus	Otu00 035	602	5,78	1,31	1,00 E-05	7,10E-04
Streptococcaceae	Streptococcus	Otu00 061	23	4,02	1,09	2,34 E-04	8,25E-03
Peptostreptococcaceae	Peptostreptococcus	Otu00 069	20	-3,48	1,13	2,16 E-03	3,67E-02
unclassified Firmicutes	unclassified	Otu00 102	10	-3,77	1,28	3,18 E-03	4,49E-02
Carnobacteriaceae	Granulicatella	Otu00 108	48	-4,61	1,37	7,53 E-04	2,03E-02
Prevotellaceae	Prevotella	Otu00 128	6	-4,41	1,40	1,63 E-03	3,15E-02

Neisseriaceae	Neisseria	Otu00 153	3	-3,60	1,13	1,52 E-03	3,15E-02
Streptococcaceae	Lactococcus	Otu00 176	5	5,77	1,72	7,68 E-04	2,03E-02
Carnobacteriaceae	Granulicatella	Otu00 386	5	3,28	1,07	2,25 E-03	3,67E-02
Carnobacteriaceae	Granulicatella	Otu00 392	6	4,89	0,99	6,94 E-07	7,36E-05
Carnobacteriaceae	Granulicatella	Otu00 399	5	3,37	1,05	1,27 E-03	2,99E-02
Pasteurellaceae	Aggregatibacter	-	59	3,55	1,12	1,49 E-03	1,07E-02
Bradyrhizobiaceae	Bradyrhizobium	-	4916	-4,14	1,09	1,51 E-04	2,52E-03
Corynebacteriaceae	Corynebacterium	-	221	-3,05	0,94	1,18 E-03	9,81E-03
Carnobacteriaceae	Granulicatella	-	10957	5,66	0,91	4,52 E-10	2,26E-08
Pasteurellaceae	Haemophilus	-	39637	-4,48	1,07	3,02 E-05	7,55E-04
Streptococcaceae	Lactococcus	-	19	6,06	2,03	2,84 E-03	1,58E-02
Neisseriaceae	Neisseria	-	828	-3,55	1,01	4,14 E-04	5,18E-03
Lachnospiraceae	Oribacterium	-	46	-2,22	0,86	9,90 E-03	4,50E-02
Peptostreptococcaceae	Peptostreptococcus	-	62	-3,76	1,24	2,53 E-03	1,58E-02
Erysipelotrichaceae	Solobacterium	-	68	-3,03	0,93	1,10 E-03	9,81E-03
Acetobacteraceae	-	-	7	4,61	1,82	1,13 E-02	4,63E-02
Bradyrhizobiaceae	-	-	7672	-3,31	1,15	4,13 E-03	3,37E-02
Burkholderiaceae	-	-	2716	-5,04	1,89	7,64 E-03	4,23E-02
Carnobacteriaceae	-	-	2430	3,70	0,91	4,49 E-05	2,20E-03
Corynebacteriaceae	-	-	302	-3,45	0,96	3,08 E-04	5,04E-03
Erysipelotrichaceae	-	-	81	-2,44	0,90	6,39 E-03	4,23E-02
Lachnospiraceae	-	-	704	-2,15	0,81	7,76 E-03	4,23E-02
Leptotrichiaceae	-	-	1362	-2,27	0,87	8,79 E-03	4,31E-02
Neisseriaceae	-	-	854	-2,74	0,88	1,78 E-03	2,19E-02
Pasteurellaceae	-	-	13860	-3,87	1,00	1,10 E-04	2,70E-03
Peptostreptococcaceae	-	-	47	-3,23	1,07	2,62 E-03	2,57E-02
**Number of ERC examinations**							

Enterobacteriaceae	unclassified	Otu00008	640	1,47	0,25	5,74E-09	6,55E-07
Carnobacteriaceae	Granulicatella	Otu00018	1650	0,51	0,12	1,02E-05	3,89E-04
Propionibacteriaceae	Propionibacterium	Otu00024	227	-0,48	0,15	1,66E-03	2,71E-02
Enterococcaceae	Enterococcus	Otu00028	42	1,01	0,28	3,28E-04	7,48E-03
Bradyrhizobiaceae	Bradyrhizobium	Otu00031	530	-0,84	0,16	1,54E-07	1,17E-05
Streptococcaceae	Streptococcus	Otu00035	602	1,41	0,22	1,11E-10	2,54E-08
Neisseriaceae	Eikenella	Otu00048	90	0,77	0,23	8,29E-04	1,45E-02
Corynebacteriaceae	Corynebacterium	Otu00071	8	0,49	0,17	3,61E-03	4,84E-02
Veillonellaceae	unclassified	Otu00074	15	-0,55	0,18	2,70E-03	3,85E-02
unclassified Alphaproteobacterium	unclassified	Otu00078	9	0,74	0,16	3,41E-06	1,55E-04
Neisseriaceae	unclassified	Otu00105	3	0,81	0,22	2,70E-04	6,84E-03
Carnobacteriaceae	Granulicatella	Otu00108	48	-0,79	0,23	6,65E-04	1,26E-02
unclassified Phylum	unclassified	Otu00221	2	-1,03	0,27	1,53E-04	4,98E-03
Corynebacteriaceae	Corynebacterium	Otu00248	2	-0,80	0,26	2,47E-03	3,76E-02
Carnobacteriaceae	Granulicatella	Otu00386	5	0,68	0,18	1,86E-04	5,31E-03
Carnobacteriaceae	Granulicatella	Otu00392	6	0,85	0,18	1,37E-06	7,79E-05
Carnobacteriaceae	Granulicatella	Otu00399	5	0,64	0,18	3,90E-04	8,07E-03
Bradyrhizobiaceae	Bradyrhizobium	-	4916	-0,80	0,17	1,86E-06	7,80E-05
Neisseriaceae	Eikenella	-	141	0,59	0,21	5,38E-03	4,44E-02
Enterococcaceae	Enterococcus	-	155	0,78	0,24	1,27E-03	1,77E-02
Chitinophagaceae	Sediminibacterium	-	249	0,42	0,15	6,34E-03	4,44E-02
Staphylococcaceae	Staphylococcus	-	65	0,50	0,17	2,50E-03	2,62E-02
Streptococcaceae	Streptococcus	-	9241	0,39	0,10	1,25E-04	2,63E-03
Actinomycetaceae	-	-	546	-0,29	0,11	1,04E-02	3,44E-02
Bradyrhizobiaceae	-	-	7672	-1,04	0,19	6,29E-08	1,04E-06
Caulobacteraceae	-	-	117	0,84	0,24	5,15E-04	3,40E-03
Comamonadaceae	-	-	2011	-0,68	0,26	8,84E-03	3,24E-02

Enterobacteriaceae	-	-	42855	1,77	0,29	6,36E-10	2,10E-08
Enterococcaceae	-	-	279	1,11	0,35	1,61E-03	8,88E-03
Lachnospiraceae	-	-	704	-0,69	0,13	2,24E-07	2,46E-06
Leptotrichiaceae	-	-	1362	-0,40	0,14	4,50E-03	1,86E-02
Propionibacteriaceae	-	-	2913	-0,63	0,18	3,66E-04	3,02E-03
Staphylococcaceae	-	-	179	0,60	0,20	2,48E-03	1,17E-02
**IBD, No -> Yes**							
Flavobacteriaceae	Soonwooa	-	7	3,44	1,04	9,33E-04	3,92E-02
Staphylococcaceae	Staphylococcus	-	65	-2,00	0,54	2,09E-04	1,75E-02
Bradyrhizobiaceae	-	-	7672	1,97	0,57	6,14E-04	1,79E-02
Pasteurellaceae	-	-	13860	-1,59	0,51	1,79E-03	1,79E-02
Ruminococcaceae	-	-	347	3,44	1,16	2,93E-03	2,20E-02
Staphylococcaceae	-	-	179	-1,99	0,62	1,40E-03	1,79E-02
**Model 3 (Run + IBD + number of ERC examinations + ERC severity score)**							
**ERC severity score**							
Streptococcaceae	Streptococcus	Otu00020	728	0,28	0,09	1,08E-03	4,35E-02
Propionibacteriaceae	Propionibacterium	Otu00024	227	0,35	0,07	7,00E-08	8,54E-06
Bradyrhizobiaceae	Bradyrhizobium	Otu00031	530	0,43	0,07	1,82E-10	4,44E-08
Streptococcaceae	Streptococcus	Otu00061	23	0,35	0,08	2,14E-05	1,74E-03
Carnobacteriaceae	Granulicatella	Otu00108	48	0,38	0,10	7,82E-05	4,77E-03
Pasteurellaceae	Aggregatibacter	Otu00157	7	0,38	0,11	2,58E-04	1,26E-02
Flavobacteriaceae	Capnocytophaga	Otu00160	3	-0,30	0,09	1,25E-03	4,35E-02
Pasteurellaceae	Aggregatibacter	-	59	0,40	0,09	4,43E-06	2,66E-05
Bradyrhizobiaceae	Bradyrhizobium	-	5252	0,55	0,08	7,51E-12	1,13E-10
Burkholderiaceae	Burkholderia	-	1766	1,18	0,18	1,14E-10	1,14E-09
Flavobacteriaceae	Capnocytophaga	-	90	-0,18	0,08	1,54E-02	4,62E-02
Corynebacteriaceae	Corynebacterium	-	221	0,25	0,07	2,83E-04	1,06E-03

Neisseriaceae	Eikenella	-	141	0,32	0,12	6,11 E-03	2,04E-02
Carnobacteriaceae	Granulicatella	-	10957	0,42	0,07	9,90 E-10	7,43E-09
Pasteurellaceae	Haemophilus	-	39637	0,33	0,08	1,17 E-04	5,02E-04
Propionibacteriaceae	Propionibacterium	-	1937	0,55	0,08	2,00 E-12	6,00E-11
Bacillales_Incertae_Sedis_XI	-	-	159	-0,16	0,05	4,54 E-03	1,61E-02
Bradyrhizobiaceae	-	-	11821	0,44	0,09	7,08 E-07	5,43E-06
Burkholderiaceae	-	-	2716	1,05	0,17	3,49 E-10	5,36E-09
Carnobacteriaceae	-	-	2400	0,46	0,07	8,91 E-12	2,05E-10
Caulobacteraceae	-	-	354	0,33	0,12	6,59 E-03	2,16E-02
Chitinophagaceae	-	-	4127	0,31	0,09	7,13 E-04	4,10E-03
Comamonadaceae	-	-	2012	0,85	0,12	5,24 E-13	2,41E-11
Corynebacteriaceae	-	-	480	0,26	0,08	1,05 E-03	5,34E-03
Lachnospiraceae	-	-	776	0,19	0,06	3,29 E-03	1,37E-02
Pasteurellaceae	-	-	13878	0,23	0,08	4,16 E-03	1,59E-02
Propionibacteriaceae	-	-	4004	0,47	0,08	1,46 E-08	1,68E-07
Ruminococcaceae	-	-	347	0,86	0,16	3,27 E-08	3,01E-07
Staphylococcaceae	-	-	179	0,36	0,10	2,02 E-04	1,33E-03
**number of ERC examinations**							
Pasteurellaceae	Haemophilus	Otu00005	2504	0,94	0,16	8,31 E-09	3,72E-07
Enterobacteriaceae	unclassified	Otu00008	635	1,37	0,23	4,07 E-09	2,43E-07
Carnobacteriaceae	Granulicatella	Otu00018	1650	0,93	0,12	5,70 E-14	1,02E-11
Propionibacteriaceae	Propionibacterium	Otu00024	227	-0,48	0,15	9,11 E-04	1,36E-02
Enterococcaceae	Enterococcus	Otu00028	42	1,13	0,29	9,43 E-05	1,69E-03
Bradyrhizobiaceae	Bradyrhizobium	Otu00031	530	-0,79	0,15	2,86 E-07	1,02E-05
Streptococcaceae	Streptococcus	Otu00035	606	0,94	0,23	5,43 E-05	1,08E-03
Corynebacteriaceae	Corynebacterium	Otu00071	8	0,49	0,17	3,30 E-03	3,93E-02
Staphylococcaceae	Staphylococcus	Otu00076	9	0,86	0,25	5,85 E-04	9,52E-03

Neisseriaceae	unclassified	Otu00 105	3	0,94	0,22	2,10 E-05	4,69E-04
Carnobacteriaceae	Granulicatella	Otu00 108	48	-0,74	0,23	1,37 E-03	1,88E-02
unclassified Phylum	unclassified	Otu00 221	2	-0,83	0,27	2,48 E-03	3,17E-02
Carnobacteriaceae	Granulicatella	Otu00 386	5	0,93	0,18	4,17 E-07	1,13E-05
Carnobacteriaceae	Granulicatella	Otu00 392	6	1,11	0,18	4,63 E-10	4,15E-08
Carnobacteriaceae	Granulicatella	Otu00 399	5	0,91	0,18	4,42 E-07	1,13E-05
Pasteurellaceae	Aggregatibacter	-	59	-0,72	0,19	1,41 E-04	2,36E-03
Bradyrhizobiaceae	Bradyrhizobium	-	5252	-0,73	0,17	2,20 E-05	6,16E-04
Enterococcaceae	Enterococcus	-	155	1,19	0,29	5,14 E-05	1,08E-03
Carnobacteriaceae	Granulicatella	-	10957	0,53	0,14	1,93 E-04	2,71E-03
Pasteurellaceae	Haemophilus	-	39637	1,11	0,17	1,78 E-10	1,49E-08
Propionibacteriaceae	Propionibacterium	-	1937	-0,53	0,16	1,05 E-03	1,10E-02
Staphylococcaceae	Staphylococcus	-	65	0,59	0,18	8,51 E-04	1,02E-02
Streptococcaceae	Streptococcus	-	9241	0,46	0,11	1,18 E-05	4,96E-04
Bradyrhizobiaceae	-	-	11821	-0,96	0,20	9,05 E-07	1,11E-05
Chitinophagaceae	-	-	4127	-0,77	0,20	1,14 E-04	6,21E-04
Comamonadaceae	-	-	2012	-1,21	0,26	3,35 E-06	3,28E-05
Enterobacteriaceae	-	-	42764	1,77	0,30	2,41 E-09	5,91E-08
Enterococcaceae	-	-	279	1,38	0,37	2,30 E-04	1,13E-03
Lachnospiraceae	-	-	776	-0,62	0,14	5,56 E-06	4,54E-05
Pasteurellaceae	-	-	13878	1,02	0,17	2,12 E-09	5,91E-08
Phyllobacteriaceae	-	-	177	-0,76	0,29	8,64 E-03	3,53E-02
Propionibacteriaceae	-	-	4004	-0,70	0,18	8,78 E-05	5,38E-04
Ruminococcaceae	-	-	347	-1,41	0,34	3,89 E-05	2,72E-04
Xanthomonadaceae	-	-	91	-0,82	0,31	8,61 E-03	3,53E-02
**IBD, No -> Yes**							
Pasteurellaceae	unclassified	Otu00 059	16	2,39	0,62	1,04 E-04	1,53E-02

Pasteurellaceae	Haemophilus	-	39637	-2,09	0,52	5,22 E-05	1,31E-03
Bradyrhizobiaceae	-	-	11821	2,31	0,53	1,11 E-05	1,65E-04
Chitinophagaceae	-	-	4127	2,33	0,53	1,21 E-05	1,65E-04
Coriobacteriaceae	-	-	87	-1,19	0,40	2,59 E-03	1,77E-02
Neisseriaceae	-	-	854	-1,10	0,41	6,98 E-03	4,09E-02
Pasteurellaceae	-	-	13878	-1,96	0,47	2,58 E-05	2,64E-04
Phyllobacteriaceae	-	-	177	2,58	0,73	3,77 E-04	3,09E-03

S1 Table. Complete list of statistically significant results. All statistically significant results from all the GLMs used in this study for differential abundance analysis, except for those associated with the sequencing run variable, which was used only for controlling purposes. **Mean abundance** = mean taxon abundance (number of sequences) across data set after normalization for sequencing depth.
